# Supplementary material for: The Concordant Disruption of B7/CD28 Immune Regulators Predicts the Prognosis of Oral Carcinomas
Source: Int J Mol Sci. 2023 Mar 21;24(6):5931. doi: 10.3390/ijms24065931 (PMC10054118; doi:10.3390/ijms24065931)
Supplement: Supplementary file 1 [file ijms-24-05931-s001.zip › ijms-2238389-supplementary.pdf]

Supplementary figures

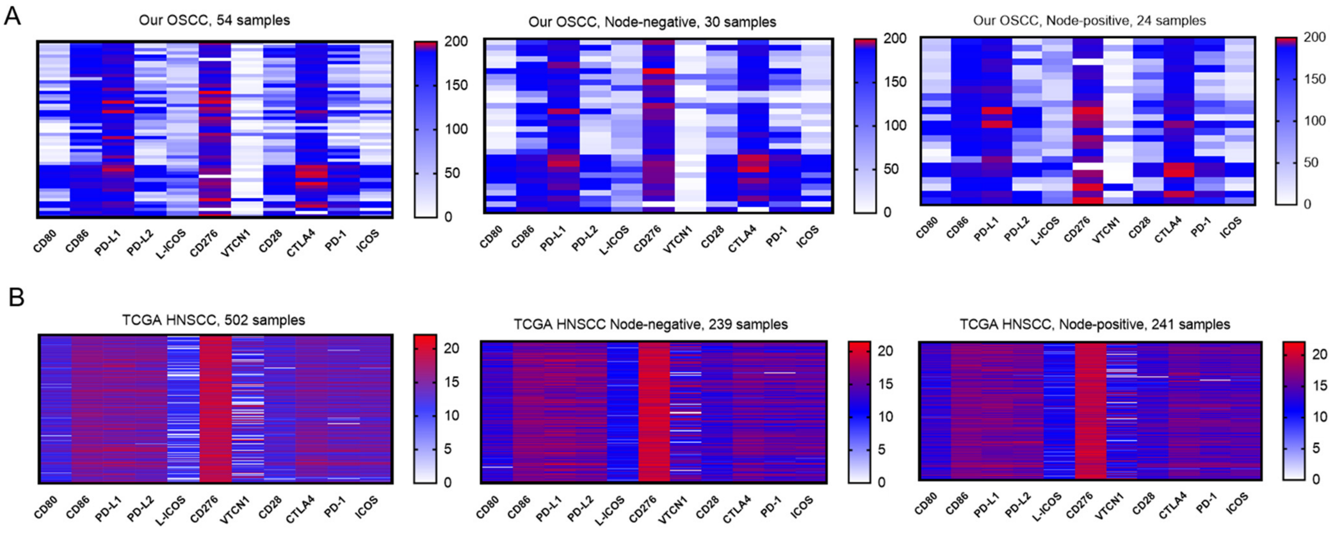

Figure S1. Heatmaps of B7/CD28 transcripts. (A) TPM of our OSCC tumor. (B) FPKM-UG of TCGA GDC HNSCC tumor. Lt, All tumor, Middle, Node-negative tumor, Rt, Node-positive tumor. Gradient bars illustrate TPM or FPKM-UQ.

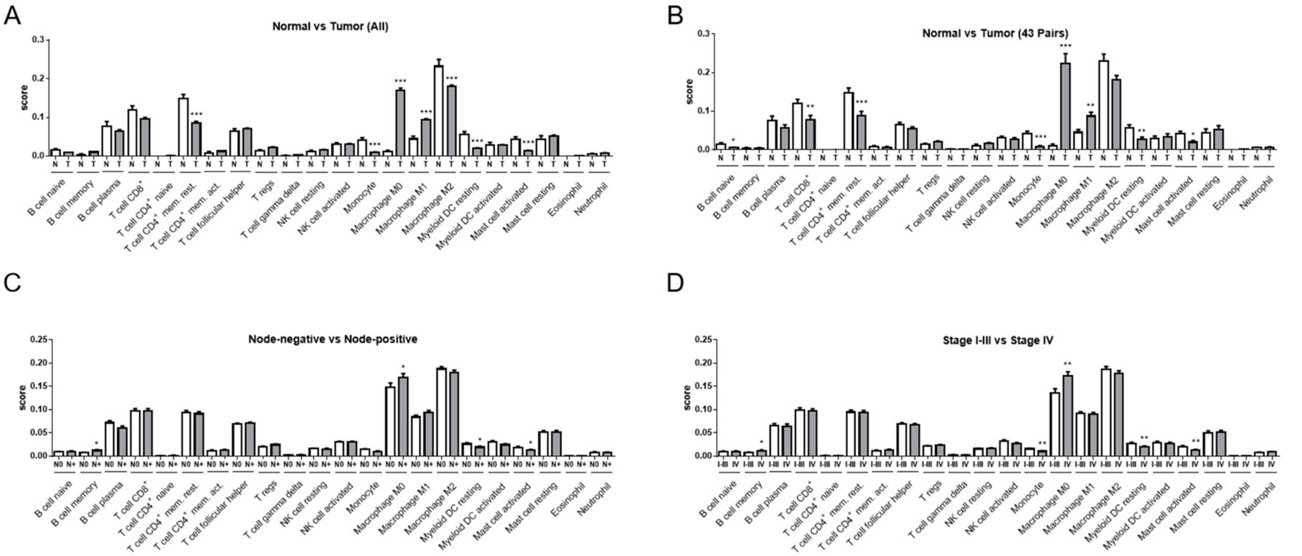

Figure S2. CIBERSORT algorithm plots to delineate the immune cell infiltration in TCGA HNSCC. (A) Normal vs tumor. (B) Paired normal vs tumor. (C) Node-negative tumor vs Node-positive tumor. (D) Stage I-III tumor vs Stage IV tumor. N, normal; T, tumor; N0, node-negative; N+, node-positive. *, ** and *** represent $p < 0.05$, $p < 0.01$ and $p < 0.001$, respectively.

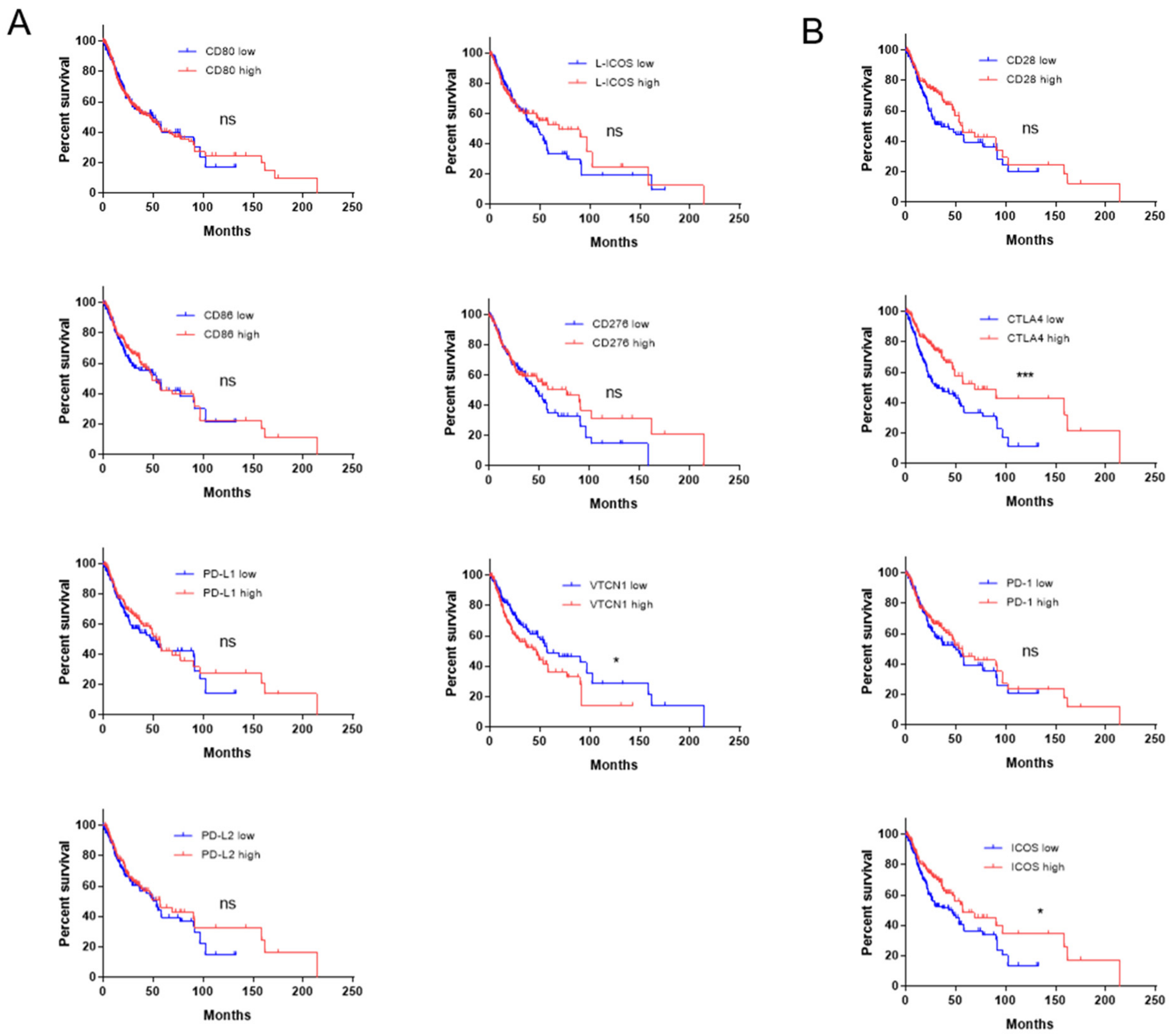

Figure S3. Survival analysis using Kaplan-Meier mode according to B7/CD28 expression in node-negative TCGA HNSCC. (A) B7 members. (B) CD28 members. *ns*, not significant; *, $p < 0.05$.

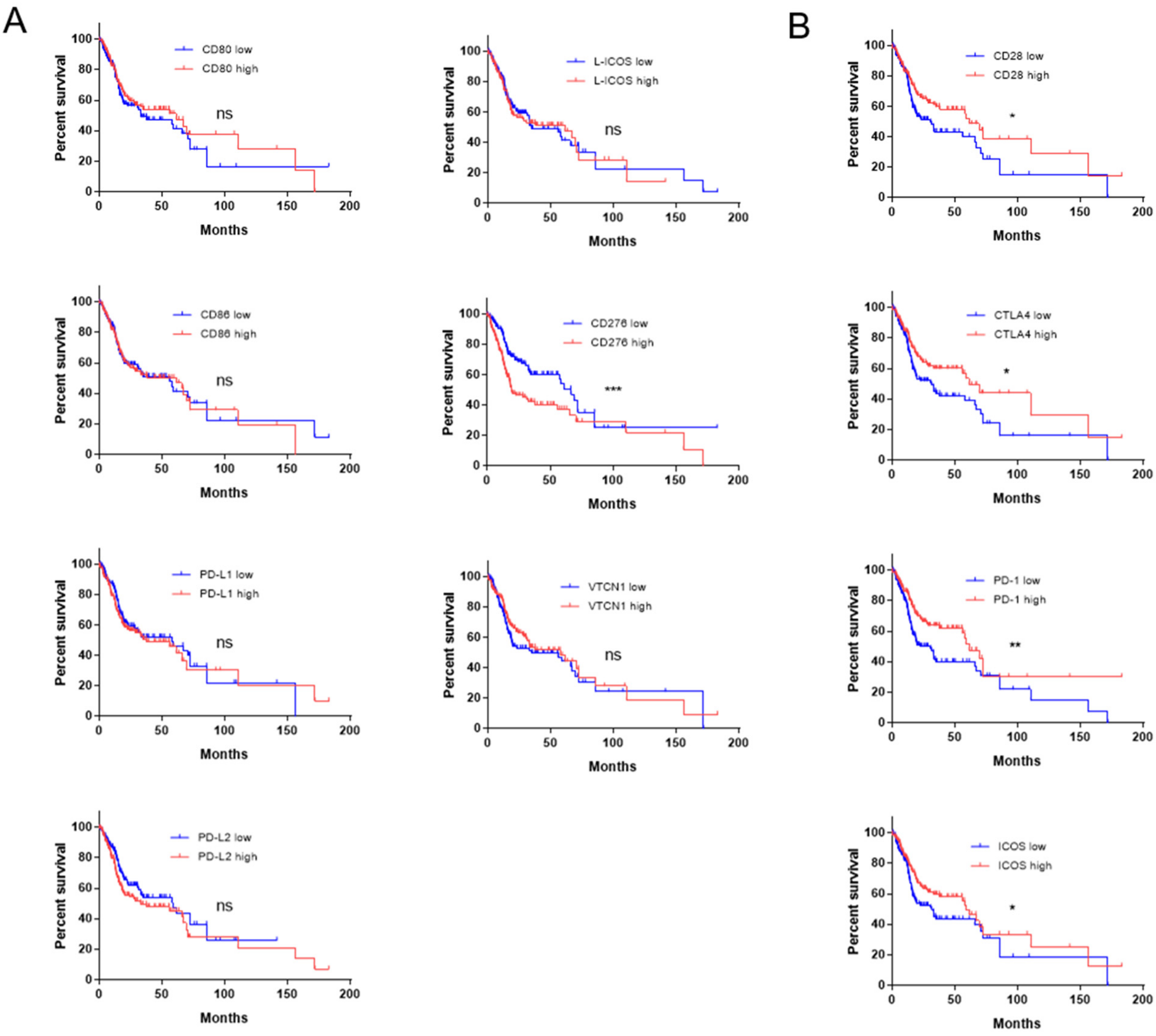

Figure S4. Survival analysis using Kaplan-Meier mode according to B7/CD28 expression in node-positive TCGA HNSCC. (A) B7 members. (B) CD28 members. *ns*, not significant. *, ** and *** represent $p < 0.05$, $p < 0.01$ and $p < 0.001$, respectively.

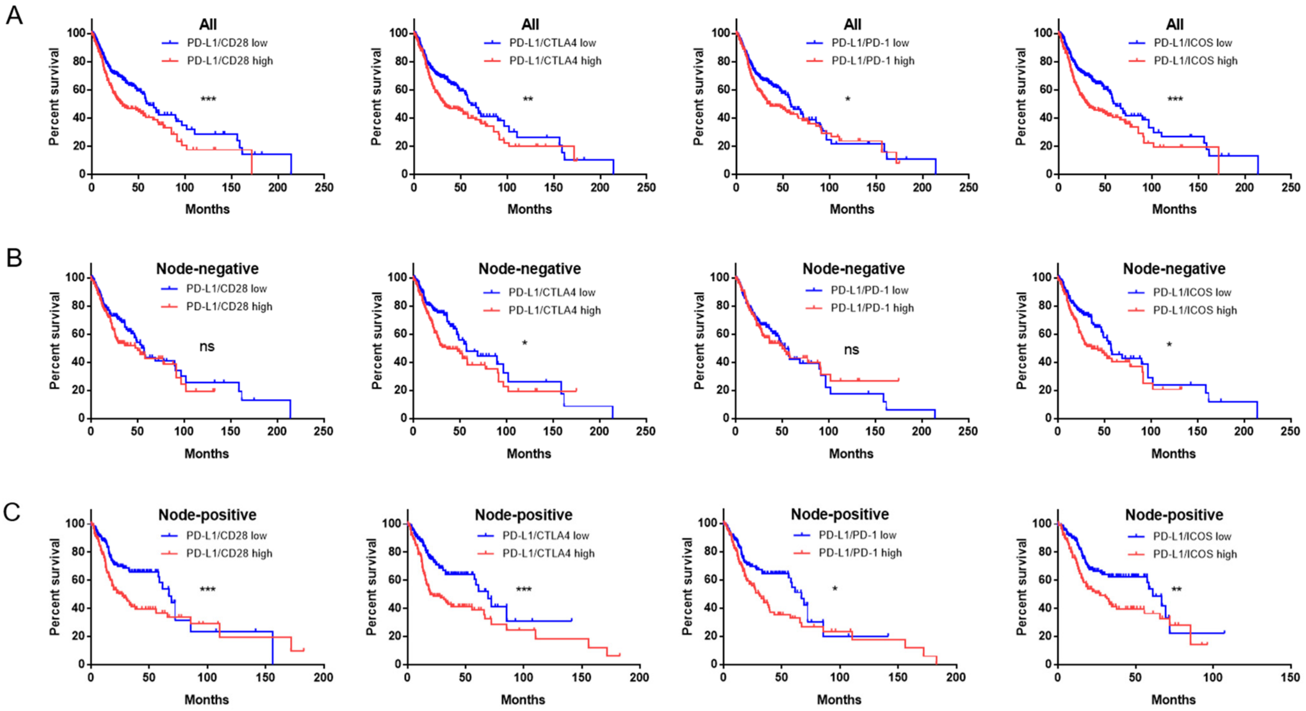

Figure S5. Survival analysis using Kaplan-Meier mode according to PD-L1/CD28 members expression in TCGA HNSCC. (A) All tumor. (B) Node-negative tumor. (C) Node-positive tumor. Lt, CD28; Lt Middle, CTLA4; Rt Middle, PD-1; Rt, ICOS. *ns*, not significant. *, ** and *** represent $p < 0.05$, $p < 0.01$ and $p < 0.001$, respectively.

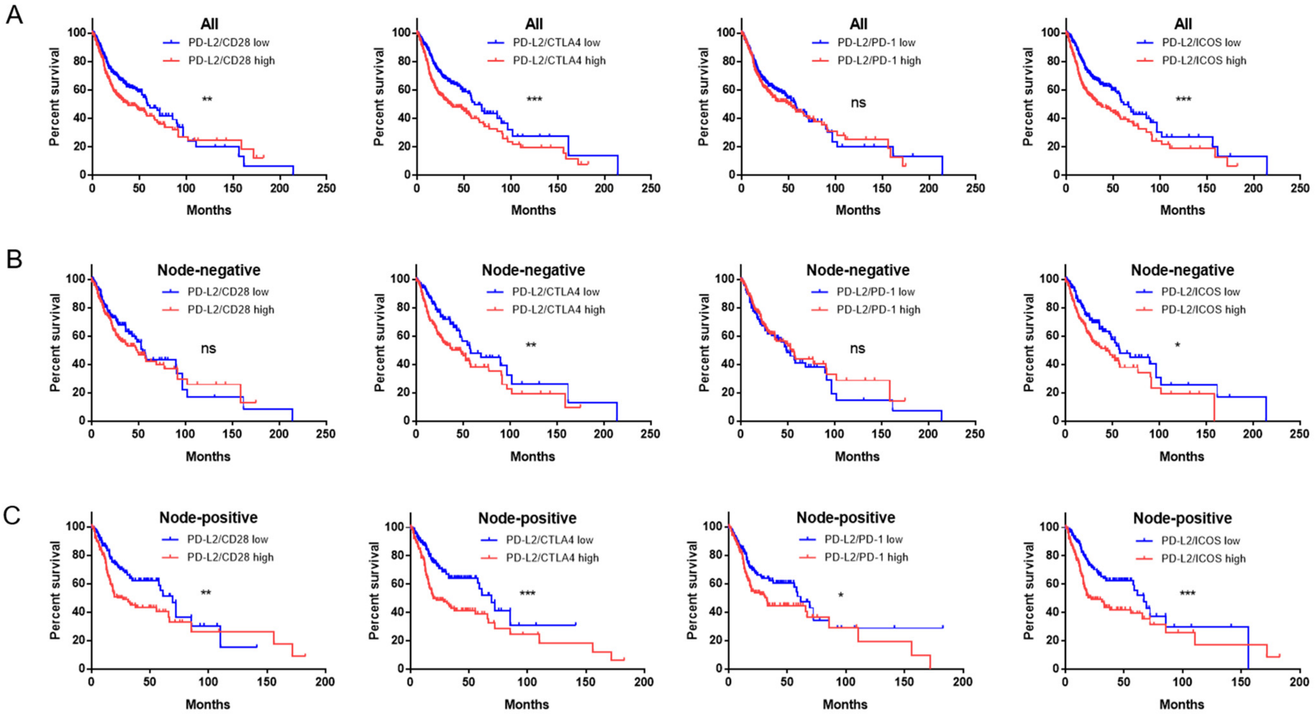

Figure S6. Survival analysis using Kaplan-Meier mode according to PD-L2/CD28 members expression in TCGA HNSCC. (A) All tumor. (B) Node-negative tumor. (C) Node-positive tumor. Lt, CD28; Lt Middle, CTLA4; Rt Middle, PD-1; Rt, ICOS. *ns*, not significant. *, ** and *** represent $p < 0.05$, $p < 0.01$ and $p < 0.001$, respectively.

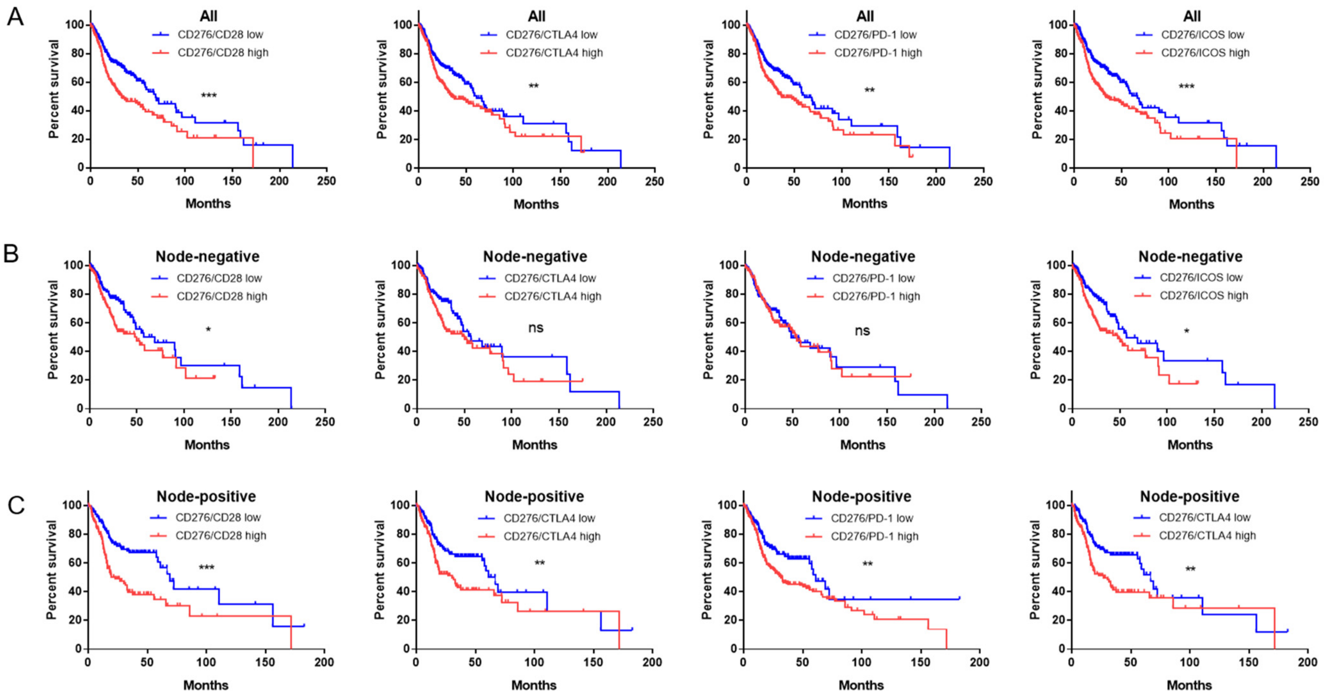

Figure S7. Survival analysis using Kaplan-Meier mode according to CD276/CD28 members expression in TCGA HNSCC. (A) All tumor. (B) Node-negative tumor. (C) Node-positive tumor. Lt, CD28; Lt Middle, CTLA4; Rt Middle, PD-1; Rt, ICOS. *ns*, not significant. *, ** and *** represent $p < 0.05$, $p < 0.01$ and $p < 0.001$, respectively.

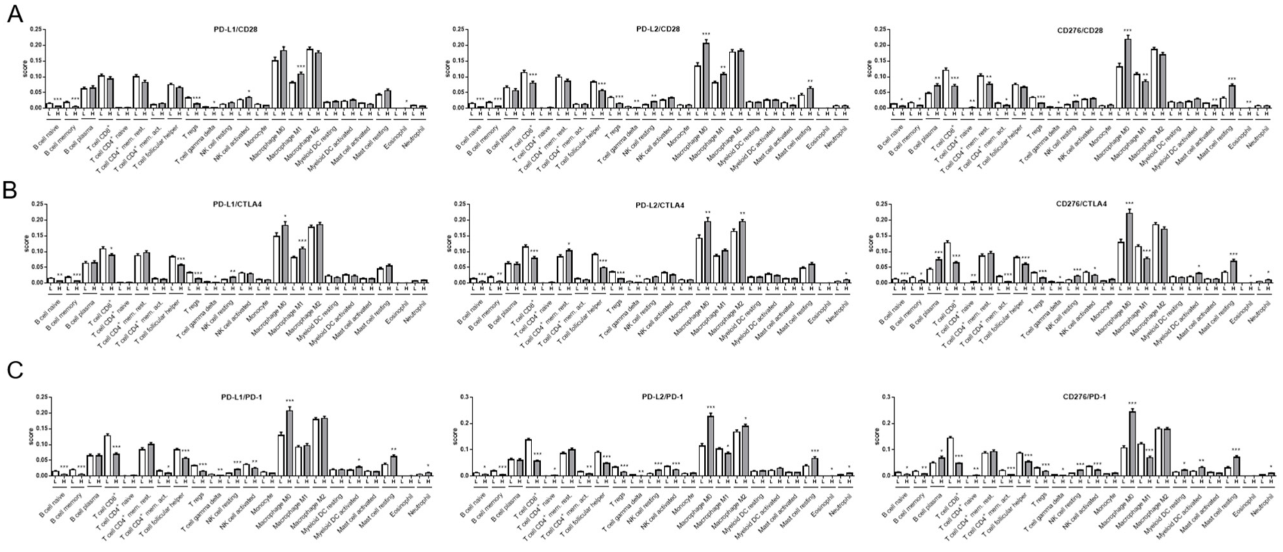

Figure S8. CIBERSORT algorithm plots to delineate the immune cell infiltration in TCGA HNSCC Node-positive tumor. (A - C) CD28 members CD28, CTLA4 and PD1, respectively. Lt. PD-L1; Middle PD-L2; Rt, CD276. L, low; H, high. *, ** and *** represent $p < 0.05$, $p < 0.01$ and $p < 0.001$, respectively.

TCGA HNSCC node-positive		B naive	B memory	B plasma	T CD8+	T CD4+ naive	T memory activated	T follicular helper	T Regs	T gamma delta	NK resting	NK activated	Macrophage M0	Macrophage M1	Mast cell resting	Neutrophil
PD-L1	CD28	***	***	ns	ns	ns	ns	ns	***	*	ns	*	ns	***	ns	ns
	CTLA4	***	***	ns	*	ns	ns	***	***	*	**	ns	*	***	ns	ns
	PD-1	***	***	ns	***	ns	*	***	***	**	***	**	***	ns	**	*
	ICOS	*	***	ns	ns	ns	ns	ns	***	*	*	ns	ns	ns	ns	ns
PD-L2	CD28	***	***	ns	***	ns	ns	***	***	**	**	ns	***	**	**	ns
	CTLA4	**	***	ns	***	ns	ns	***	***	**	ns	ns	**	ns	ns	*
	PD-1	*	***	ns	ns	***	**	***	***	**	***	***	***	*	***	ns
	ICOS	ns	**	ns	***	ns	ns	***	***	ns	ns	ns	***	ns	ns	*
CD276	CD28	***	*	**	***	**	*	ns	***	*	**	ns	***	**	***	ns
	CTLA4	***	*	***	***	**	***	***	*	*	***	*	***	***	***	*
	PD-1	***	**	*	***	**	***	***	***	*	***	***	***	***	***	*
	ICOS	ns	ns	***	***	**	**	***	***	ns	**	ns	***	***	***	ns

Figure S9. Summary of the common immune cell population in TCGA HNSCC Node-positive tumor. The data in Figure S8 are organized to show events repeatedly appear for more than four times. Grey boxes, adaptive immune cells; Green boxes, innate immune cells. The differential densities of blue colors in boxes indicate the different degrees in decreasing of cell population, while the differential densities of red colors in boxes indicate the different degrees in increasing of cell population. Note that CD276/CD28 members group displays remarkable aberrances in plasma cell, T CD4+ naïve cell and M1 macrophage in relation to PD-L1/CD28 members group or PD-L2/CD28 members group. *ns*, not significant. *, ** and *** represent $p < 0.05$, $p < 0.01$ and $p < 0.001$, respectively.
